# European Countries' Different Legal Orientation About End-of-Life Issues in Patients Affected With Neurological/Psychiatric Diseases: Does Italian Law n.219/2017 Provide Adequate Options for This Fragile Category of Patients?

**DOI:** 10.3389/fpsyt.2021.675706

**Published:** 2021-09-24

**Authors:** Nicola Di Fazio, Silvia Romano, Zoe Del Fante, Paola Santoro, Vittorio Fineschi, Paola Frati

**Affiliations:** ^1^Department of Anatomical, Histological, Forensic and Orthopaedic Sciences, Sapienza University of Rome, Rome, Italy; ^2^Istituto di Ricovero e Cura a Carattere Scientifico (IRCCS) Neuromed Mediterranean Neurological Istitute, Pozzilli, Italy

**Keywords:** dementia, end-of-life, assisted suicide, advance treatment provisions, euthanasia

## Introduction

In the last 20 years, the international panorama has been affected by several legislative openings regarding the “end of life.” This sensitive and delicate issue, in fact, has played a pivotal role in the ethical–legal debate for many years, leading to law promulgation in many countries of the European Union. Following the example of many progressive countries, Italy has recently promulgated a law addressing this topic. More specifically, law 219/2017 focuses on fundamental aspects of patients' will, from informed consent to refusal of treatment and advanced treatment provisions, with particular reference to incapable patients (1). In Italy, at present, law 219 supports incapacitated patients with various figures according to the severity of illness. On the other hand, with regard to assisted suicide, the Italian Constitutional Court requires full capacity of the person, precluding this possibility to incapable patients.

## European Situation

In the European context, the initial opening toward assisted suicide sees Switzerland as a leading country. Swiss penal code, enacted in 1918, already recognized the possibility of resorting to assisted suicide, punishing only those who applied it for personal purposes. Assisted suicide refers to the active conduct of a patient who voluntarily causes his death by taking lethal medical substances. Nowadays, Switzerland is the only country where assisted suicide procedure is allowed, not only for residents but also for foreign patients.

In fact, many persons travel to a country with the principal aim of ending their life. This phenomenon is unique to Switzerland, and it is defined as “suicide tourism.” Indeed, it has been increasing over the years and is still growing (2, 3).

In 2002, in Belgium, decriminalization of euthanasia was witnessed through the definition of specific evaluation criteria to identify eligible patients. The term euthanasia indicates any act aimed at intentionally causing the death of a person, following his explicit will. Passive euthanasia occurs whenever death results from treatment omission (i.e., life-saving treatments), while active euthanasia, on the other hand, refers to death induced by the action of third parties (i.e., drug administration).

Among the eligibility criteria for euthanasia, identified by Belgian legislation, there are age of the patient (minors are permitted to request euthanasia only in case of physical illnesses), mental competence, voluntary and repeated request in the absence of external pressure, incurable somatic or psychiatric pathology, and presence of constant and unbearable physical or psychological suffering (4). Procedural criteria are also envisaged, aimed at verifying the conscious and unchangeable will of the patient. The acquisition of several physician opinions along with compliance to a 1-month waiting period between a written request for euthanasia and execution of the procedure is envisaged (5).

In the same year, the Termination of Life on Request and Assisted Suicide Act was promulgated in the Netherlands. It provided for the use of such procedures in compliance with specific eligibility criteria, similar to those applied in Belgium (voluntary request by the person, intolerable and deprived suffering, prospects for improvement, absence of therapeutic alternatives, awareness of one's prognosis, and acquisition of the opinion of several accredited specialists) (6).

In 2005, passive euthanasia was legalized in France. Subsequent attempts at greater openness in this sense were rejected, recognizing only the possibility for doctors to apply deep terminal sedation.

In April 2009, Luxembourg established that doctors who practiced euthanasia and assisted suicide were not criminally or civilly prosecutable, by the provisions of the law.

Countries such as Spain, Sweden, England, Hungary, and Norway allow the use of passive euthanasia only in restricted circumstances (7).

Furthermore, in Italy, there has been a jurisprudential opening on end-of-life issues, through the enactment of law 219/17 about informed consent and advanced treatment provisions, even though several previous sentences on the matter. In fact, this law introduces advanced treatment provisions (ATP), statements relating the patient's consent or dissent to a specific future health treatment (8). ATP can be drawn up by mentally capable adults. They can express their will in anticipation of a possible future inability to self-determine. The same law equates artificial nutrition and hydration to medical treatments and introduces the possibility of patients' refusal. It also defines the prohibition of therapeutic persistence, however, providing for the use of palliation in terminal patients to alleviate their suffering.

Another innovation introduced by this law is the possibility to plan individualized treatments based on the patient's value system (9). Such choices should be made while the patient's autonomy and decision-making capacity are still fully intact. Although far from progressive European countries' rulings, law 219/17 represents an important regulatory advance in the Italian normative system, making it the first “end-of-life law” in Italian legal history.

Another recent innovation in Italy is the possibility of resorting to assisted suicide. The Constitutional Court with sentence no. 242 of 2019 recognized this possibility only under certain conditions, considering not punishable those who facilitate the execution of a person kept alive by life-sustaining treatments and suffering from an irreversible pathology, but fully capable of making free and informed decisions. These conditions and methods of execution should be verified by a public structure of the national health service, after consulting the territorially competent ethics committee. People with mental disorders are therefore not included in this possibility.

## Discussion

In those European countries where euthanasia is already legal, the number of patients suffering from neurological/psychiatric diseases who request to benefit from this therapeutic option is constantly increasing. This perspective raises numerous ethical, philosophical, and scientific concerns (10). In fact, unlike somatic pathologies, psychiatric and neurological ones, by their very nature, could compromise the patient's ability to self-determine and therefore to exhibit valid consent.

Consequently, a debate on the legitimacy of resorting to euthanasia/assisted suicide in patients suffering from dementia takes on particular weight (11).

The term dementia refers to a condition of chronic and irreversible cognitive impairment, generally associated with advanced age or, rarely, genetic mutations. The prevalence of dementia in industrialized countries is about 8% in the over 65s and rises over 20% in people aged over 80 (12). Due to the increasing average age, dementia diseases are frequently diagnosed.

Moreover, modern personalized medical approaches aim to predict individual susceptibility to neurological and psychiatric pathologies, to identify their response to pharmacological treatments (13).

The clinical onset of the disease is characterized by cognitive or behavioral symptoms, most times mimicking psychic pathologies. In the advanced stages of the disease, the motor sphere is involved, compromising the patient's ability to independently feed and carry out daily life activity. Death usually occurs from cachexia and infective complications.

Depressive symptoms associated with dementia, particularly evident in the early stages of the disease, may include suicidal ideation. Therefore, death request in the presence of depressive symptoms requires correct classification, since it could constitute a psychopathological expression rather than an effective, conscious desire (14). However, scientific studies show that suicide prevalence in patients affected by dementia is not higher than in the general population. This could be related to the progressive loss of insight that accompanies the deteriorating evolution of the dementia disease (15), thus making the patient less able to understand its own health condition and prognosis. Indeed, the severity of dementia inversely correlates with suicidal risk (16).

Further problems concerning the possible application of ATP in people suffering from dementia are represented by a lack of topicality of the disease when drawing up those provisions.

Although dementia represents a terminal condition, cognitive deterioration occurs slowly, making it difficult to identify a suitable evolutionary timing, thus reflecting in complex advance planning of care. As previously mentioned, in advanced stages, the ability to self-determine is lacking in most cases, due to disease characteristics. Therefore, the hypothesis of an early-stage ATP drafting would not be based on adequate awareness of the disease. Although the patient suffering from initial symptoms of dementia may project himself into future worsened conditions, he will not be able to fully understand those cognitive, emotional, and behavioral alterations inevitably associated with a different stage of the disease. In addition, we must remember that dementia diagnosis is rarely made in disease-free or extremely early stages. More specifically, only in the case of family genetic disorders genetic screening can allow early diagnosis in the absence of typical clinical manifestations.

In dementia early stages, before the patient becomes totally incapacitated, after assessment of his decision-making capacity, any wishes about the type of treatment should be stated and results of the assessment should be recorded.

In those rare cases in which clinical diagnosis occurs in the initial phase of the disease, as already mentioned, depressive symptoms are generally expressed. By its very nature, the coexistence of suicidal ideation should be carefully evaluated to distinguish it from the normal presentation of a mood disorder. To this end, it could be useful to stratify patients according to disease severity, by administering psycho-cognitive tests. This would result in a greater understanding of various domain impairments, necessary to valuate expression of a valid consent. To this effect, psychiatric guidelines have been drawn up in the Netherlands in order to objectively frame patients requiring assisted suicide/euthanasia procedures suffering from psychiatric pathologies (17). Should this possibility be implemented in Italy, the collaboration of the neurological and psychiatric team would be desirable for the evaluation of dementia and mood disorders.

Currently, in Italy, law 219/17 only provides for the possibility of refusing care, nutrition and hydration, and request for palliative care. More specifically, the law defines the pivotal role in any medical activity of informed consent, which should be given after thorough discussion about the specific situation/disease carried out with healthcare professionals. New provisions introduced by this law are shared care planning and advanced directives (18). Shared care planning refers to a long-term disposition given by the patient about his/her future therapeutic strategies whenever chronic diseases are involved. To achieve such an objective, patients and doctors should successfully collaborate, sharing medical knowledge and making well-pondered decisions. Advance directives, on the other end, define therapeutic strategies chosen by the patient in provision for future inability to formulate consent on any treatment. As for its specifics, the patient plays a fundamental role while defining its future diagnostic and therapeutic options, which doctors are required to comply with.

In the case of dementia, there are no specific effective therapies, and assistance is limited to the treatment of associated complications. Moreover, treatment consists in counteracting bedding complications, terminal phase commonly witnessed in these cases, through ventilatory assistance, nutrition, and artificial hydration. The person's concrete liability would therefore be exclusively that of supportive treatment refusal in the terminal phase of the disease, using previously signed ATP. This raises questions about ATP's concrete appropriateness in guarantying personal dignity protection (19). Consequently, a gray area is identified in which the person does not own the right to choose to end his life, while maintaining those fundamental characteristics of dignity. Patient's chances would therefore be limited to the possibility of accelerating death through suspension of terminal care, when already experiencing late phases of illness and suffering (20, 21).

In conclusion, although law 219/17 arises from the need to put individual dignity at the fore, in compliance with the self-determination principle constitutionally protected in the Italian legal system, there are still numerous questions about its concrete applicability. Although more easily applicable in somatic pathologies, characterized by exclusive deterioration of the person's physical abilities, its application in the psychiatric/neurological field appears more complex. Loss of the patient's cognitive abilities and his capacity to self-determine represents a major limitation of voluntary refusal of treatment. When the person is willing to express his consent to treatment through ATP, the challenge lies in the clear definition of a limit beyond which the disease has already compromised his mental faculties. In the light of the ever-increasing number of people suffering from dementia, it would be advisable to identify precise guidelines by means of a specialist multidisciplinary team, promoting the use of psychometric assessment tools aimed at a proper evaluation of each patient.

## Author Contributions

NDF and PF conceptualized the study. SR, ZDF, and PS wrote, reviewed, and edited the manuscript. VF supervised the study. All authors contributed to the article and approved the submitted version.

## Conflict of Interest

The authors declare that the research was conducted in the absence of any commercial or financial relationships that could be construed as a potential conflict of interest.

## Publisher's Note

All claims expressed in this article are solely those of the authors and do not necessarily represent those of their affiliated organizations, or those of the publisher, the editors and the reviewers. Any product that may be evaluated in this article, or claim that may be made by its manufacturer, is not guaranteed or endorsed by the publisher.
